# Strengthening the capacities of a national health authority in the effort to mitigate health inequity—the Israeli model

**DOI:** 10.1186/s13584-016-0077-4

**Published:** 2016-08-15

**Authors:** Tuvia Horev, Shlomit Avni

**Affiliations:** 1 Department of Health Systems Management, Guilford Glazer Faculty of Business and Management, Ben-Gurion University of the Negev, P.O.B. 653, Beer-Sheva, 8410501 Israel; 2 Strategic and Economic Planning Administration, Israel Ministry of Health, Jerusalem, Israel; 3 Department of Politics and Government, Ben-Gurion University of the Negev, Beer-Sheva, Israel

**Keywords:** Health equity, Health inequality, Disparities, Public policy, National health policy, Interventions

## Abstract

The need for a national policy to mitigate health inequity has been recognized in scientific research and policy papers around the world. Despite the moral duty and the social, medical, and economic logic behind this goal, much difficulty surfaces in implementing national policies that propose to attain it. This is mainly due to an implementation gap that originates in the complex interventions that are needed and the lack of practical ability to translate knowledge into practices and policy tools.

The article describes the Israeli attempt to design and implement a national strategic plan to mitigate health inequity. It describes the basic assumptions and objectives of the plan, its main components, and various examples of interventions implemented. Limitations of the Israeli policy and future challenges are discussed as well. Based on the Israeli experience, the article then sketches a generic framework for national-level action to mitigate inequalities in health and in the healthcare system. The framework suggests four main focal points as well as an outline of the main stakeholders that a national policy should take into consideration as agents of change.

The Israeli policy and the generic framework presented in the article may serve researchers, decision-makers, and health officials as a case study on ways in which prevalent approaches toward the issue of health inequality may be translated into policy practice.

## Background

Health inequality is associated with political, social, and economic phenomena that various countries address in consideration, *inter alia,* of values and norms that are typical of the society in which the system operates.

The need for a national-level policy for mitigating socioeconomic inequality in general, and health inequality in particular, has been recognized in scientific research and policy papers by many health organizations and countries around the world [1–6]. In 2008, a WHO committee released a landmark report that stressed the impact of the social determinants of health disparities and delineated principles for action to tackle them [7]. The 2011 Rio Declaration [8] expressed the WHO member states’ commitment to fighting health inequity by addressing the social determinants of health.

Various countries have been acting for several decades to develop and implement policies that have the mitigation of health inequality as their goal [9]. Since the beginning of the current century, there has been evidence of an upturn in the efforts pledged to this cause. Some countries are taking structured and consistent action at the national level; others are applying focused interventions at lower levels.

Despite the moral duty and the social, medical, and economic logic behind narrowing health inequality among states and among social groups within them, the implementation of national policies that propose to mitigate inequality and attain meaningful outcomes has proven very difficult. The literature suggests a plethora of factors that impede the attainment of an official, consistent, coherent, and effective commitment to the reduction of inequality. They include lack of political will and power to make thorough changes in social norms, i.e., those outside the healthcare system, that generate disparities in health, “poor governance” for equity in health through action on social determinants [10], a lack of incentives that might prompt various players to mitigate inequalities, the existence of disincentives to the desired outcome, and a shortage of ideas and new ways of doing things (“doing more of the same”). In addition, governments and stakeholders within the healthcare system point to an implementation gap occasioned by the complex interventions that are needed to contend with health inequality and the lack of practical ability to translate the knowledge about the etiology of health disparities into practices and policy tools [10–12]. High-visibility approaches such as “action on the social determinants of health,” “securing political commitment,” the “life-cycle approach,” “health equity in all policies and activities,” “proportionate universalism,” “involving local people” and “multi-sectorial cooperation” [9, 13] are described in theory but rarely translate into concrete policies, action items, and tools [14].

Israel, like many other developed countries, has socioeconomic disparities. Poverty rates among households in Israel, after the effects of transfer payments and taxes, are nearly the highest among OECD member states. According to the 2012 OECD average (for 32 states) the average poverty rate after taxes and transfers was 0.109 while Israel’s average was 0.184. The only country with higher poverty rates then Israel was Mexico (0.186). The Gini index of inequality in income distribution also portrays Israel in a worse light than most OECD member states. The 2012 Gini OECD average (for 31 states), post taxes and transfers, was 0.308, while Israel’s was 0.371. The only countries with a higher Gini than Israel were- the US (0.39), Turkey (0.402) and Mexico (0.457) [15, 16].

Several studies conducted in Israel revealed wide socioeconomic inequalities in health and in Israel’s healthcare system. For many years, the Israel Ministry of Health (MoH) dealt with their implications by means of local and sporadic interventions as opposed to a structured national plan. In 2010, the ministry undertook to tackle health inequity as one of its strategic objectives for the years 2011–2014. Thus, during the first four years of implementation, the state intervened in various aspects of activities and policies and invested approximately 2.2 billion ILS ≈ 0.6 billion USD- in infrastructure and manpower in the periphery and 1.6 billion ≈ 0.4 billion USD - on reduction of economic and cultural barriers to health-care services, just to name two activities [17].

This article describes the basic assumptions that guided MoH in preparing its plans, presents the conceptual framework, and describes the focal points of intervention and the policy tools that the government used. It also addresses additional initiatives of relevance in narrowing health disparities that the government promoted but were not part of the aforementioned plan. Then, based on the Israeli experience, it proposes a generic national (and regional) concept for intervention, demonstrating ways in which prevalent approaches toward the issue of health inequality and ways of dealing with them may be translated into policy practice.

We hope that the Israeli experience as well as the generic conceptualization of the steps taken in Israel at the national level will contribute to policymakers’ and government officials’ knowledge and supply an additional perspective that, combined with their own experience and knowledge, will broaden their ability to confront the complicated task of transforming knowledge on health disparities into national-level policies and practices.

### Laying the foundations for a national plan to mitigate health inequity

As a result of several publications [18–24] that preceded an upturn in public awareness, MoH launched a comprehensive effort to address health disparities in 2009 by establishing a dedicated unit for this purpose. In 2010, as it planned its strategic objectives for the coming years, it designated diminishing health inequality as a strategic objective for 2011–2014 [25, 26]. The Economics and Health Insurance Division (subsequently upgraded to the Strategic and Economic Planning Administration) assumed responsibility for spearheading the effort, in conjunction with the other Ministry divisions. The idea was to engineer specific system-level changes and integrate gap-narrowing activities into the annual work plans of relevant Ministry divisions as well as those of the health funds (public healthcare providers akin to HMOs) and other actors in the healthcare system.

#### Strategy selection

The basic assumptions that guided MoH before implementation were four: Social and economic gaps in Israel will not be eliminated in the near future, meaning that MoH will have to deal continually with the health manifestations and outcomes of socioeconomic disparities; some inequalities in the healthcare system are not related to socioeconomic determinants; MoH and the healthcare system have a relatively limited influence on upstream interventions that may impact the social determinants; and concentrating only on a downstream approach will deliver relatively little efficacy at the national level. Based on these assumptions, MoH decided to focus its main efforts on areas within its purview and control in order to leverage change nationally that would strengthen the capacity of the healthcare system to cope with inequality (i.e., focusing on a “midstream approach”). Even so, MoH did not abandon its efforts to influence social determinants of health as well (an “upstream approach”). Finally, it was assumed that the intervention would yield fruitful health outcomes only in the long term. Therefore, in the first few years, goals would be measured by process indicators rather than by health-outcome indicators.

#### Defining the target population

MoH decided to combine an across-the-board policy (for the entire social gradient) with activity centering on target population groups. This decision reflected approaches expressed in the literature, stressing the need for an intervention across the entire socioeconomic gradient with proportional emphasis, including specific resource allocation, on disadvantaged groups [9].

MoH’s main activity in tackling disparities in the availability of healthcare services centered chiefly on the country’s geographic periphery, whereas activities to overcome economic barriers and enhance the cultural competence of healthcare organizations and staff targeted socioeconomically disadvantaged populations irrespective of their geographical location. As mentioned, all this would be augmented by national-level interventions across the socioeconomic gradient.

#### The policy concept

On the basis of these assumptions and insights, MoH chose six strategic goals for the years 2011–2014: narrowing gaps in economic access to healthcare services; alleviating the effect of disparities in accessibility and quality of healthcare services due to cultural barriers; enhancing the availability of adequate and high-quality professional medical personnel in peripheral areas; improving physical infrastructures in peripheral areas; assuring the availability of relevant data and information on interventions to reduce health inequality; and assuring the existence of correct incentives and auditing tools for effective implementation so to encourage various stakeholders to invest more in activities that would promote and ensure health equality.

### Implementation: focal points of intervention

In this section, we give several examples of interventions toward each of the six strategic goals that MoH chose for the years 2011–2014 1. We then describe interventions aimed at the social determinants of health (SDH) that were promoted during that period concurrent with progress toward the six strategic goals.

### Narrowing gaps in economic access to healthcare services

Action to remove economic impediments to crucial healthcare services falls into two main categories: relief in copayments for services provided under the National Health Insurance Law and the inclusion of essential medical services and products, until recently funded wholly by households, in the basic statutory “basket” of services.

#### Relief in copayments

An example of this first area of activity was the cancellation of a user charge that mother-and-child clinics had been paying for prenatal and neonatal services such as pregnancy follow-ups, child-development checkups, and vaccinations. Charges attending to these preventive services may impair access, especially for members of socioeconomically weak population groups. Another example was the reduction of the copayment for generic medicines covered by National Health Insurance from 15 percent to 10 percent of the prices shown on the MoH reference price list.

#### Inclusion of services in the statutory basket

An example of the second area of activity concerns the inclusion of pediatric dental care in the basket of services covered by National Health Insurance. This might be considered as an example of an intervention that, while aimed at the entire social gradient, impacts the narrowing of inter-group disparities. This step, taken at MoH’s initiative, was followed by requisite legislative changes once the government approved it. Thus, since July 2010, preventive and preservative dental care for children, until then largely privately funded and delivered, was phased into National Health Insurance on the basis of age groups. In this manner, by January 2016, all children in Israel up to age fourteen were eligible for this care through the public system. The services covered included the possibility of preventive dental care with no copayment and a set of preservative (restorative) dental treatments (e.g., dental fillings) with relatively low copayments. A research conducted in 2013 found that 64 % of all children aged 2–11 (age cohorts that are eligible to receive dental treatments under the national health insurance) and 70 %–79 % of children between 6–12 year of age, visited a dentist in the past year. Only 3 % of the parents reported to have given-up on dental treatment for their child (treatment recommended by a physician), the main reasons being that the child was afraid and/or uncooperative (price was not given as a reason). No disparities were found among children from different socio-economic groups in contrast to wide disparities revealed among children 12-16 years, who were not eligible for treatments under the national health insurance law. However, the research shows that Arabs of low socioeconomic status still underutilize services relative to Jews of similar status (although the study did not find the difference between groups to be significant) [27].

### Narrowing health disparities due to cultural barriers

#### Setting national standards

In an attempt to overcome disparities occasioned by cultural barriers, the MoH Director General distributed in 2011 a circular to all main healthcare-service providers, public and private, that set standards and norms in cultural and linguistic access to healthcare services. In response, HMOs and other organizations (e.g., government-owned hospitals) promoted actions in line with the guidelines newly set forth. Examples of such actions include the appointment of an official in charge of cultural and linguistic access in each establishment; translation of brochures, forms, medical information, and Web sites; and the tailoring of information and interventions to patients’ different cultural backgrounds and needs.

#### Translation services

Since 2013, MoH has been contracting with an outsourcer that runs a telephone call center providing real-time translation services into Arabic, Russian, Amharic, and French for people who undergo community and inpatient medical care. The center started as a pilot project in 2013 reached full speed in 2014. Several HMOs offer these services as well. Between 2013 and 2015, the number of calls to the translation service in Russian grew by 464 percent, Amharic by 75 percent; and Arabic by 35 percent (There are more than 1,000 calls per month and the number is constantly and rapidly growing) [28].

#### Enhancing the cultural competence of healthcare staff

MoH offered courses to train officials as cultural competence officers and to instructors in charge of training medical teams on the issue. In 2014, MoH began and completed the development of a training kit to give organizations tools to train their healthcare providers and enhance their cultural competence. The kit includes theoretical material and information about cultural competence tailored to different cultures in Israeli society, as well as lesson plans and video simulations of various medical encounters that pose challenges stemming from patient–provider cultural differences. The simulations demonstrate issues such as reliance on a religious authority in making health-related decisions, the structure of the family unit and its implications for the use of healthcare services among various population groups, and assessment of and coping with pain and states of morbidity in different cultures, to name only a few. The kit is meant for use by instructors in group workshops run by healthcare organizations and by lecturers in academic institutions that train students for work in the healthcare field.

### Enhancing the availability of medical personnel in peripheral areas

#### Expanding training capacities in peripheral areas

A critical and highly meaningful step toward the development of medical human infrastructures in peripheral areas was taken with the establishment of a medical school in the Galilee town of Safed (Northern District) four years ago. This is enormously significant not only due to the added increment of medical personnel that it will provide at the national level but also for the development of healthcare services and human resources in the Galilee, where physicians have been in short supply relative to the rest of the country. This initiative was the result of the cooperation between MoH and strategic partners in the government (e.g., the Negev and Galilee Development Ministry) and the Council for Higher Education. At the present writing, 386 students are enrolled in the new school (in their last years of training), 17.4 percent of medical students countrywide at the same stage of their training. Training of nurses in schools in the periphery has also accelerated in recent years (the annual registration of new nurses climbed from 929 in 2010 to 1,974 in 2013) [29].

#### Encouraging physicians to move to the periphery

In 2009, as the result of an MOH initiative and staff work with the Commissioner of Wages at the Ministry of Finance, an inter-ministerial committee was set up to recommend ways to attract medical personnel to peripheral areas. Hardships in the field were studied, lessons from previous measures to attract people to the periphery by HMOs and others were learned, and effective steps to leverage change were identified. As the committee wound up its work, the physicians’ collective agreement with the government came up for renewal and negotiations with the Israel Medical Association (IMA) began. The very representatives of the ministries who sat on the committee were the ones who conducted these negotiations on behalf of the government. The comprehensive collective agreement that emerged at the end of the talks included two meaningful incentives to attract personnel to the periphery: a wage increase for doctors who accept jobs in hospitals in peripheral areas and grants for specialist trainees who choose to train in areas of short supply and in peripheral hospitals, provided they perform a minimum term of service in the periphery. The accord also gave the healthcare system a major increase in job slots for physicians (roughly 1,000), with the periphery given priority. Today, peripheral areas are showing a steady upturn in physicians relative to 2008–2010 (averages of 2.3 physicians per 1,000 residents in the north and 3.0 in the south in 2012–2014 as against 1.6 and 2.2, respectively, in 2008–2010) [30]. The change traces to an increase in the rate in the periphery and a decrease in the center of the country.

#### Incentives for nurses who move to the periphery

In 2009, several mother-and-child clinics in the southern Bedouin sector had to shut down for lack of nurses to fill available positions. In 2011, incentives were introduced for nurses who work in the Southern District generally and among the Bedouin particularly. As a result, all nurses’ posts in mother-and-child clinics in the south were filled by 2011–2012.

#### Adequate representation

Action was taken to train healthcare personnel who belong to target population groups. One class of nurses from the Bedouin sector, held under the auspices of Ben-Gurion University (degree track), graduated in 2014; another class (certification track) that began its studies in 2014 is due to graduate in 2016 [29].

Due to a shortage of professionals in certain paramedical fields in the Southern District and the lack of training programs in occupations such as speech therapy and occupational therapy at degree-awarding institutions, MoH asked the Council for Higher Education (CHE), which oversees such institutions, to approve the establishment of training programs in these occupations, under the auspices of a university or college in the south that the Council would find appropriate. After mulling the request, the Council authorized two accredited colleges in the south to present it with detailed programs for its approval. One such program (in speech therapy), submitted and pending approval, would encourage Bedouin students in the south to enroll in the courses by providing them with professional and financial support and stewarding. Thus, MoH is attempting to attain two goals related to the narrowing of disparities—expanding the supply of human resources in these occupations in the Southern District and creating an infrastructure of professionals in the Bedouin sector.

### Improving physical infrastructures in peripheral areas

#### Physical infrastructures

Israel faces a challenge in regard to differences among geographic areas in hospital beds. The ratio of general hospital beds to population has been declining in recent years in all areas other than Jerusalem and the Northern District. In accordance with an agreement between Moh and the Finance Ministry, an increase of some 1,000 hospital beds, about half earmarked for hospitals in the periphery, was authorized for the years 2011–2017. Additionally, over a three-year period (2010–2013), licenses were issued for the operation of MRI machines in the north and south as well as a mobile MRI. The addition of a line accelerator for a hospital in the north (for use in treating cancer patients) was also approved. The establishment of twenty-five specialist units for four hospitals in peripheral areas was authorized—78 percent of all such units approved in 2009–2012. In addition, a special budget was earmarked for the establishment of ten urgent-medicine facilities in peripheral localities, with funding divided among the Ministry of Health, municipal authorities, and the HMOs [31].

### Assuring the availability of relevant information to improve the coping capacity of planners and executives

The purpose of this objective was to assure the availability for planners and executives at both the government and the regional level of relevant information on states of health by socioeconomic indicators, access to and availability of services, and “good practices” and effective interventions for the mitigation of health inequality.

#### Establishing a dedicated national research unit on health inequality

The development of a statistical data and information infrastructure that specializes in and focuses on health inequalities is essential as a basis for action to mitigate inequality and enhance the ability to monitor and develop mechanisms of improvement, follow-up, and re-evaluation. Accordingly, MoH set up and funded a unit, based in the Gertner Institute for Health Policy and Epidemiology, to produce research, information, and data for use in monitoring health inequality using uniform and standard methodology. Now that such a unit exists, it is possible to gather and analyze information on health disparities and monitor trends over time. In the course of 2012, researchers at the Institute, in conjunction with MoH, drew up a five-year work plan that was approved by the MoH Director General and has been implemented gradually in ensuing years.

#### Analysis of data on disparities in quality indicators

The National Program for Quality Indicators in Community Healthcare, led by MoH and the Israel National Institute for Health Policy Research (NIHP), is a well-known project that has drawn praise in OECD reviews of Israel’s healthcare system [32]. The program turns out analyses of data on disparities in quality indicators of healthcare services parsed by age groups and socioeconomic status, comparing low-ranking groups (defined by exemption from copayments) with others. Over the years, the program has shown steady improvement in its abilities; its next annual reports will include relevant information by an additional socioeconomic variable based on geographical units. Its reports are accessible to the public.

#### A platform for information-sharing—the annual national conference

An annual conference organized by MoH, titled “The Healthcare System Tackles Inequity,” provides fertile soil for information exchange and sharing among healthcare-system stakeholders concerning measures that may be used to mitigate inequality. Senior managers of HMOs, hospitals, and MoH gather to discuss and present their successes and challenges. In the course of the conference, the HMOs are also required to describe their activities and targets for promoting equality. The presentation of achievements during the previous year and plans for the coming year also promotes competition among HMOs and provides a continuous incentive for improvement, creativity, and sharing of knowledge. Six conferences have been held thus far.

#### Publication of an annual report

The annual national report, titled *Coping with Health Inequality*, is published by MoH ahead of the national conference and includes data on MoH and HMO activities to narrow gaps. Hospitals are also invited to contribute to the report; occasionally they, too, describe what they have done to mitigate disparities. The annual reports, along with MoH publications on health inequality and ways of dealing with it [30, 33–36] as well as other MoH publications on additional aspects of this issue [37, 38] over the years, have kept the struggle against health inequality on the agenda, furthered the sharing of relevant information, and incentivized and promoted competition so to continuously improve organizational action against health inequality.

#### Information on health rights for citizens and executives

In 2012, MoH published a booklet based on diverse statutory sources that summarizes clearly and comprehensively the full set of healthcare-service entitlements of thirty special-status groups such as the elderly, children with special needs, persons who lack resident status, and prisoners and detainees [39]. The booklet was distributed to all healthcare organizations in Israel and was translated into Arabic and Russian. MoH also set up a portal and a call center that enhances access to information about entitlements and encourages consumers to exercise them. These initiatives reflect an effort to enhance the transparency of specific healthcare services to which underserved groups are entitled by law and to present the services in a way that overcomes jargon and language barriers.

### Developing incentives and auditing tools for stakeholder motivation

Successful implementation of national interventions depends, among other things, on various incentives and their impact on stakeholders’ willingness to cooperate and become agents of change. Several examples follow:

#### Prospective payments to encourage HMO’s to invest in the periphery and to empower insured persons who live in the periphery

An example is the capitation formula by which most of the basic budget for national health insurance is allocated to the HMOs. The formula serves two main goals: to predict HMOs’ expenditures with the best possible fit to members’ characteristics and to encourage HMOs to invest in populations or areas chosen by MoH. Until 2009, the only variable taken into account in the formula was the number and age composition of each HMO’s members. This served as a proxy for the use of healthcare services and a predictor of HMO expenditure for delivering the services covered by national health insurance. From 2010 onward, the formula was revised by adding two components—gender and distance of place of residence from central Israel. The latter was meant to encourage investment in the geographic periphery. Since this is a prospective payment that overweights HMO members who live in the periphery, it was expected to stimulate competition among the HMOs for these members and encourage investment in services for them as a way to retain their membership.

#### Retrospective payments

Another intervention created economic incentives in the form of financial support of HMOs contingent on meeting specific targets. It was part of an agreement that governs HMO subventions under criteria set forth by MoH. The criteria included investment in health-promotive intervention programs for targeted population groups in geographical and social peripheries as well as investment in infrastructure in remote areas. The HMOs were allowed to choose the intervention and the location on their own (within a framework defined by MoH). Since 2012, when the incentive was first introduced, activity of this kind has grown steeply. The annual number of interventions reported by HMOs and approved by MoH under the subventioning criterion that entails the promotion of health and healthy behavior among target populations (as defined by MoH) increased from 93 in 2012 to 460 in 2013 and 435 in 2014. The number of interventions implemented to improve health-services infrastructure in the periphery grew from 90 in 2012 to 199 in 2013 but receded to 101 in 2014 (because the review process was toughened) [30]. Additional interventions that were not approved under the subventioning criteria were reported and are also important.

Other incentives, allocated by the MoH public-health division, encourage HMOs to emphasize physical activity, smart nutrition, and maintaining blood-sugar balance among members of high-risk groups—with higher incentive scores awarded to HMOs that aimed their interventions at targeted socioeconomic groups.

#### Auditing tools

MoH developed and revised the auditing tools that it uses in its periodic inspection visits to HMOs and hospitals in order to evaluate their activities in narrowing health disparities and their compliance with its circular on cultural competence. Reports on routine inspections of public clinics and hospitals by MoH representatives are uploaded to the MoH web site for viewing by the public and by managers in the healthcare system.

### Actions addressing the social determinants of health

Parallel to the six strategic objectives mentioned above, MoH, together with other partners in and outside the government, has pursued initiatives to impact the social determinants of health (SDH). Although the main focus of this article is on interventions within the remit of a national health authority, a brief presentation of several examples of such initiatives is relevant to the topic.

#### Public participation in policy formulation and interventions

MoH established a round table for brainstorming with several civil-society organizations, in which regular discussions are held on ad-hoc issues concerning underserved groups in order to involve the community in policy decisions. Civil-society partners were also involved in designing the aforementioned training kit. Thus, this product, although spearheaded by the Ministry and facilitated by professionals, was planned in close cooperation with some forty content consultants including representatives of various population groups, NGOs, academics, and caregivers in relevant fields.

MoH also created a role for the public in a review of its policy on promoting the integration of Ethiopian Israelis. The process, undertaken pursuant to a government resolution concerning the outlining of a policy that would optimize these citizens’ social integration, was directed by an interministerial forum of twelve government offices headed by the Director General of the Ministry of Immigration and Immigrant Absorption. The process owes its uniqueness to the regular consultations that took place with representatives of the Ethiopian-Israeli community in joint round tables, separate round tables for each government office, and an online forum.

#### Poverty and health

MoH cooperated with the Ministry of Labor and Social Affairs (today the Ministry of Welfare and Social Services) by participating in a “war-on-poverty” committee set up by the Minister of Labor and Social Affairs. In this capacity, MoH contributed to the establishment of a subcommittee on poverty and health. The committee published recommendations on encouraging access to and availability of healthcare services for impoverished population groups and on mitigating health disparities that trace to income and class factors.

### Proposed conceptual framework for a national action plan

Here, basing ourselves on the process that MoH led, as described above, and on the experience gathered during initial stages of implementation, we propose a conceptual framework for a national action plan to mitigate disparities in health and in the healthcare system. It should be emphasized that although the proposed framework centers on interventions within the remit of a national health authority, it does not obviate the importance of efforts to impact the social determinants of health as well.

### Focal points of intervention

**Fig. 1:**
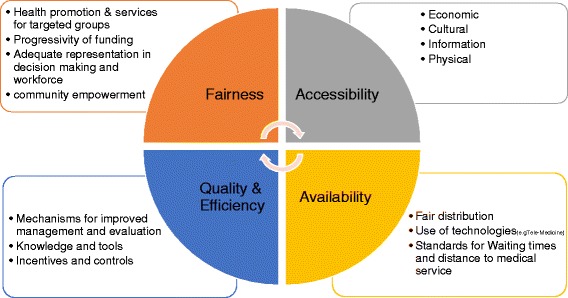
Focal points of intervention

Figure 1 (below) presents a concept on which decision-makers and planners may call. The figure emphasizes four main focal points of intervention at a national or regional level and gives examples of possible interventions in each of the four focal points.

Possible interventions for narrowing disparities in “accessibility” to health services, in accordance with the UN International Covenant on Economic, Social and Cultural Rights, relate to four main areas of involvement: economic access to services (e.g., lowering copayments so that members of socioeconomically disadvantaged populations do not forgo crucial services including those covered by national health insurance), cultural access (interventions that may improve the cultural competence of services in accordance with the recipient’s cultural background, including the provision of translation services), access to information (interventions meant to make information more transparent, available, and comprehensible to the public, in the sense of increased accessibility to relevant data and information and the simplification of professional terminology and jargon) and physical access (relating to the accessibility of services for persons with disabilities and members of vulnerable population groups.)

At the core of the “availability” focal point are interventions that enhance equality in the distribution of services (community and inpatient infrastructures and personnel), establish and enforce norms in regard to waiting times, and develop technologies such as telemedicine services that may bridge gaps between center and periphery and support the chronically ill and persons who have disabilities or lack family or social support.

The “quality and efficiency” focal point relates to tools and incentives for insurers and service providers that encourage investment in the development of high-quality, accessible, and available services specifically in geographical peripheries, in socioeconomically disadvantaged areas, and among target groups. Creating and disseminating data on trends in disparities and ways to deal with them is an additional area that calls for intervention. Furthermore, investing in the improvement of management processes in the targeted areas, enhancing the efficiency of the interventions applied there, and building mechanisms for evaluation and improvement are crucial so that, wherever an activity is found to be ineffective, corrective measures to attain the hoped-for results will be taken. Economic and non-economic incentives are also crucial for the creation of motivation and commitment to action among stakeholders and actual investment in infrastructure and interventions.

The “fairness” focal point concerns activities that enhance the fairness of the healthcare system’s funding and activities. Examples are assuring greater progressivity in funding; adequate representation of diverse cultural backgrounds and socioeconomically weak population groups among professionals, providers, and managers in the healthcare system; mechanisms for fair representation of diverse populations in decision-making; and action to promote health and medical services that target specific groups.

### Agents of change

A national health authority cannot tackle inequality on its own; it must collaborate with various outside stakeholders. Some such stakeholders may act to narrow health disparities by force of conviction and belief; others, however, need encouragement and incentives to do so and should receive them. The national health authority may consider different groups of stakeholders as potential agents of change in mitigating health inequalities and should sort various policy tools and incentives in order to find those that may be effective vis-à-vis each of the relevant agents.

**Fig. 2:**
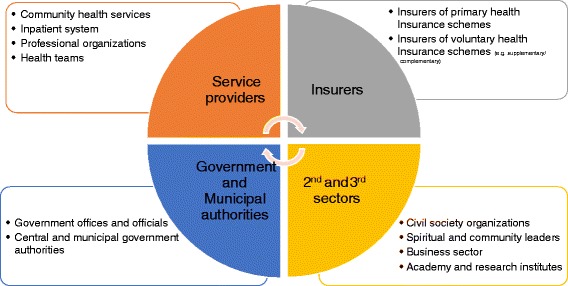
Agents of change

Figure 2 (below) sets forth the conceptual basis of this issue. It parses the agents into categories: four principal categories and main specific agents in each category. Although different national healthcare systems exhibit different structures, degrees of government involvement, and dependence on agents, we believe that, due to the diversity of the stakeholders, a national healthcare system should give special consideration to several groups of agents in particular:

Insurers—generally speaking, health insurers are significant actors in any healthcare system. However, the extent of governmental intervention and the policy tools used in the case of insurers that supply the statutory package of services or primary-private health insurance schemes, whether for profit or non-for profit (mutuality organization), is different compared to the kind of interventions and policy tools that should be used when the insurer provides a voluntary private health-insurance scheme (e.g. duplicate private health insurance, as well as complementary or supplementary to the primary scheme).

Service providers—the national health authority may interact with community caregivers and the inpatient system, for example, to help them improve their cultural competence, encourage them to extend service to faraway or less-desirable localities, and so on. Professional associations may be meaningful agents of change because they strongly affect the authority’s ability to offer incentives to the healthcare workforce working in the periphery or with disadvantaged groups and educate health-care workers on the issue of health inequality.

The 2^nd^ and 3^rd^ sectors - Civil society, comprising the social, spiritual, and opinion leaderships of specific groups along with nonprofit service providers and social change organizations, is an additional important agent. Large businesses from the private sector often engage in social involvement; they too may be agents of change among their employees and/or among communities for which they offer social/philanthropic activities; they may also be potential subventioners. Academia and research institutes may assist a national health authority by gathering and analyzing data for use in evaluating long-term trends in the extent of inequality and the impact and efficiency of interventions implemented. Additional agents of change may be umbrella organizations in academia and healthcare training institutes in charge of accreditation, quality assurance, and financing. Their involvement may be helpful, for example, in expanding training venues in order to meet needs in specific geographical regions that are short on professional human resources; they may also abet the promotion of adequate representation of various groups in healthcare occupations and in courses that train future caregivers to deal with cultural differences.

Central- and municipal-government authorities—Cooperation with relevant government offices, such as those in charge of welfare and education, can encourage joint interventions that may impact the social determinants of health (e.g., health and prevention education). Municipal authorities are also potential agents of change; in some countries, they are key actors in the field.

## Discussion

This article sets forth the Israeli national plan to reduce health inequality. Instead of presenting the full minutiae of the plan, it lays out the conceptual framework and gives examples of the main focal points of intervention and the policy tools that are used to assimilate the policy chosen. The conceptual framework emphasizes the need to adopt a “midstream approach” and to concentrate mainly on aspects that fall within the responsibilities and the purview of a national health authority. Its aim is to strengthen the capacities of the healthcare system in coping with health disparities, focusing on six main themes and envisioning collaboration with agents of change by means of leverages and policy tools that will encourage them to act in accordance with the chosen policy.

The article challenges statements in the literature about factors that impede the attainment of an official, consistent, and coherent commitment to the reduction of inequality in healthcare systems. The Israeli case demonstrates that a national policy and an action plan adopted by the national health authority can enhance the capacity to cope with inequalities and ensure greater access to healthcare services by eliminating economic barriers, improving providers’ cultural capacity, encouraging investments in peripheral areas and among target population groups, enhancing fairness through interventions that target health promotion and health services for underserved groups, promoting adequate representation of target groups in healthcare professions and managerial positions, boosting community empowerment; and further developing the quality and efficiency of services provided in peripheral areas and/or for target population groups at large.

The Israeli case shows that when a national health authority takes a leading position, it can create the needed political will, carry out the requisite budget earmarking, establish incentives for stakeholders, and promote abundant creativity and ideas for intervention to mitigate health inequalities. The foregoing account shows, in broad terms, that a national authority responsible for the population’s health at the federal or national level has much room to address inequalities in the healthcare system without abandoning its commitment to continual efforts to deal with the social determinants of health inequality.

Although each country’s healthcare system is unique, the main focal points of intervention presented here (e.g., accessibility, availability, quality and efficiency, and fairness), are common, as may be the principal stakeholders. Interventions and policy tools, however, should be selected very carefully in accordance with the values, structure, targets, and characteristics of each national system.

In view of the wide socioeconomic disparities that exist in Israel, however, MoH faces substantial challenges in its attempts to narrow health gaps. Given the long-term impact of any policy that tackles health inequality on health outcomes, no quick win is expected. Therefore, when one deals with health inequality, as with other public policies, sustaining an ongoing national plan such as the one described in this article in a reality of regular change in governments poses a challenge of its own—one that should not be taken for granted. As a matter of fact, in 2013, following elections in Israel and the establishment of a new government and the appointment of a new deputy minister of health 2, MoH dropped the mitigation of health disparities as a specific strategic goal; instead, it integrated this objective into the overall goal of “health promotion.” During a window of opportunity that opened between 2011 and 2013, however, MoH launched several initiatives that may serve as a sustainable platform for further progress in the future. They include, among others, establishing a dedicated unit for reducing inequality, creating a specialized research center, concluding a collective agreement between a professional association and the state that empowers physicians in the periphery, revising the capitation formula in ways that enhance health equity, setting standards and norms on cultural competence, producing a training kit to enhance providers’ cultural competence, and setting up a translation call center; to name only a few.

In the Israeli case, additional challenges persist. They concern, in the main, leveraging the problem of inequity into a pan-governmental responsibility, improving horizontal and vertical integration (“horizontal” referring to cooperation among government ministries such as Welfare and Social Services, Education, the Economy, and Finance, and “vertical” meaning the co-optation of municipal authorities and district health centers as important players), and recruiting the public and civil society for the cause. Reinforcement of the public-health system, including an increase in public funding of healthcare, is also needed. Progress in these matters would also facilitate meaningful action to mitigate the effects of the social determinants of health.

### The need for further research

Some of the activity reviewed in this article, took place at a time that one may describe as a ‘window of opportunity’. It was just then that the senior professional stakeholder in this field at the Ministry of Health (MOH) initiated a meaningful change that fit the perspectives of the director general and acting minister (deputy minister at the time). Thus the cause was defined as an MOH strategic objective, by force of which policy was made and a dedicated plan was applied. Such a ‘window of opportunity’, however, may of course close or change; this happens when a change of Government results in the appointment of new ministers and director general or when public atmosphere changes. Indeed, after the Government, the Minister of Health, and the Director General were replaced, MOH did continue to promote action on health inequality in Israel but no longer defined it as one of its strategic objectives.

This study focused less on the process of making policy in the aforementioned field than on describing and analyzing the policy chosen, its rationale, and the interventions that the Government carried at the time. Nevertheless, the reasons and circumstances that pried the ‘window of opportunity’ open deserve to be researched. The nexus of an initiative that successfully implements a new public policy and astute exploitation of a window of opportunity is recognized in the scientific literature [40]. Thus, the Israeli story may serve as a test case for research that will make it possible to analyze, for example, the circumstances that catapulted the question of coping with health inequity to the decision-makers’ agenda specifically in 2009; why the decision-makers adopted it; what significance this window of opportunity held; and whether policy in this field can be changed and applied on a similar scale under other circumstances. Each of these questions is an important one that merits thorough additional research. Generally speaking, it is noteworthy that the disparities described in the article between vast knowledge about health inequality and the mechanisms that produce it, and actual practice in dealing with it, probably relate not only to an implementation gap, tracing to the lack of practical proposals, but also to proposals on how to surmount obstacles that sometimes prevent a policy from being implemented—including barriers associated with detecting a ‘window of opportunity’ that opens or closes in response to diverse political, social, or economic circumstances. Thus, research on this topic, including the Israeli case among processes in other countries, may be meaningful in identifying the factors that inhibit or strengthen commitment to and implementation of a thorough, sustainable national policy to reduce health inequality.

## Conclusion

A national health authority and other players in the healthcare system, operating by themselves, may be able to make only a relatively weak impact on the social determinants of health. Nevertheless, the very broad latitude that they enjoy within their official remit may result in a substantial positive impact on narrowing health inequalities. By commission and omission, the healthcare system can aggravate inequality; to the same extent, it can mitigate it. Even as the system acts within its purview, however, action on the social determinants should be taken in concert with others to set a long-term effort in motion. To bring this about, cooperation among ministries and higher government prioritization of the treatment of socioeconomic gaps will be needed.

It is premature to predict whether the processes launched in Isreal will persevere and be sustainable over the years and how they will impact health indicators in the long term. This matter deserves additional follow-up and research that will monitor outcomes in health and other domains, it being borne in mind that access to high quality healthcare services and lowering economic and cultural barriers are values that justify themselves even if their immediate impact on health outcomes is not proven.

The focal points of intervention presented in this article are not unique to Israel. In each country however, they may be differently expressed. Generally speaking, a national health authority can maximize its capacities to act within its own locus of control and find a way to carry out interventions that are geared to the narrowing of health disparities in accordance with the structure of its healthcare system, its challenges, and its dominant values—all of which, without relinquishing leadership in the struggle against the social determinants of health inequity.

## Footnotes

1 The information to be presented is based on [10] and gives only a few examples of the interventions that have been implemented.

2 Due to internal political circumstances, members of Israel’s ultra-Orthodox parties do not hold ministerial portfolios but serve as deputies. In the new government, MoH has been run by the ultra-Orthodox representative, Rabbi Yaakov Litzman, as a deputy minister, with Prime Minister Benjamin Netanyahu officially holding the portfolio. Due to an appeal to the supreme, court, in the current government Raabbi Litzman is the Minister of Health.
